# A two-tier model of abduction: a unified framework for perception and emotion

**DOI:** 10.3389/fpsyg.2025.1732693

**Published:** 2025-12-12

**Authors:** Jian Sun

**Affiliations:** School of Educational Sciences, Guangxi Minzu Normal University, Chongzuo, China

**Keywords:** abductive insight, abductive reasoning, percept, perceptual judgment, emotional construction, predictive processing

## Abstract

**Introduction:**

A central tension exists within Charles S. Peirce’s philosophy of mind: while he characterizes perception as an “extreme case of abduction,” its involuntary nature seems to exclude it from the realm of reasoning. This paper aims to resolve this tension and develop a unified framework for understanding perception and emotion.

**Methods:**

We propose a two-tier model of abduction, distinguishing between abductive insight (the spontaneous, non-volitional generation of a hypothesis) and abductive reasoning (its conscious, conceptual endorsement). This theoretical model is integrated with the Predictive Processing framework, which provides a mechanistic account of abduction as hierarchical prediction error minimization.

**Results:**

The model demonstrates that the formation of the perceptual gestalt (the percept) constitutes abductive insight, while the conceptual categorization of that gestalt (the perceptual judgment) constitutes abductive reasoning. This framework is successfully extended to the domain of emotion, showing that Barrett’s theory of constructed emotion instantiates the same architecture: core affect serves as abductive insight into interoceptive signals, and emotional categorization acts as abductive reasoning that makes affective states intelligible.

**Discussion:**

The two-tier model of abduction successfully resolves the Peircean tension and provides a unified account of perception and emotion. This synthesis contributes to philosophical psychology by revealing how conceptual operations (abductive reasoning) shape affective experience. Furthermore, it suggests practical implications for emotion regulation, pointing to interventions that can target either the level of core affect (abductive insight) or emotional categorization (abductive reasoning).

## 1 Introduction

Charles S. Peirce’s theory of abduction confronts a foundational paradox. While he explicitly characterizes perceptual judgments as an “extreme case of abductive inferences” (Peirce, 1931–1958, CP 5.181), thereby situating perception within the domain of abduction, he simultaneously restricts “reasoning” to consciously controlled and voluntary operations, thus excluding the involuntary nature of perceptual processes. This generates a central theoretical question: how can perception be both a form of abduction and yet fail to qualify as reasoning? To address this, it is necessary to closely examine the distinction between abduction as a broad class of hypothetical inference and abductive reasoning as its conscious form. This paper takes that distinction as its starting point, establishing a unified framework for understanding two fundamental domains of mind: perception and emotion.

The solution, I propose, lies in a fundamental distinction within abduction itself: a two-tier model that cleaves the process into (1) abductive insight—the spontaneous, unconscious generation of a hypothetical explanation (e.g., the formation of a perceptual gestalt), and (2) abductive reasoning—the conscious, deliberate formation of a hypothetical explanation (e.g., the perceptual judgment). The primary novelty of this model is its unified explanatory power. I demonstrate that this distinction not only addresses the problem in Peirce’s account of perception but also extends elegantly to the domain of emotion, offering a parallel account of emotional construction.

This two-tier model serves as the central integrator, weaving several core frameworks into a unified account by assigning each a distinct yet complementary role.

First, Peircean abduction provides the fundamental logical structure. Originating from the philosopher Charles S. Peirce, abduction is the process of forming explanatory hypotheses. Its most influential classic form is: “The surprising fact, C, is observed; But if A were true, C would be a matter of course; Hence, there is reason to suspect that A is true” (CP 5.189). This paper builds upon this by distinguishing between unconscious abductive insight and conscious abductive reasoning.

Second, the Predictive Processing framework supplies the mechanistic account of how this logic is implemented in the brain. Predictive Processing (PP) views the brain as a hierarchical prediction engine that continuously generates top-down predictions about sensory inputs. The difference between prediction and actual input is prediction error, which the brain fundamentally seeks to minimize (Clark, 2013). I employ PP as the neuro-computational mechanism that implements Peircean abduction. A growing body of empirical work continues to support this framework (Clark, 2025; Fernández Velasco and Loev, 2024; Luppi and Smith, 2024), providing a robust empirical foundation for the arguments advanced here.

Third, Barrett’s Theory of Constructed Emotion offers the domain-specific psychological model that my framework directly instantiates. This theory, pioneered by Lisa Feldman Barrett, argues that emotions are not innate reflexes but are constructed in the moment by the brain. This construction involves two key components: (1) core affect (a basic, continuous feeling state derived from internal body signals), and (2) the categorization of this affect into a specific emotion concept (e.g., “anger,” “fear”) using prior experience and cultural context. This model maps these two components directly onto the two tiers of abduction.

Furthermore, the Peircean distinction between the percept and the perceptual judgment provides the crucial phenomenological bridge that illustrates this two-tier structure within the familiar domain of perception.

With these frameworks in mind, I now turn to elaborating my two-tier model of abduction in three parts. Section 2 elaborates the two-tier model and demonstrates its application to perception. Section 3 extends the model to emotion, integrating the theory of constructed emotion. The conclusion synthesizes the findings.

## 2 Abduction and perception

This part of my argument aims to accomplish two primary goals: to clarify the Peircean question outlined in the introduction by introducing my two-tier model of abduction, and to demonstrate its application to the domain of perception.

### 2.1 Abduction

In the philosophy of Charles S. Peirce, abduction is the process of forming an explanatory hypothesis—it is the only logical operation that introduces new ideas. Peirce’s theory of abduction contains a fundamental tension that my two-tier model aims to resolve: it encompasses perceptual judgments as an “extreme case” of abduction while excluding them from “reasoning” proper due to their unconscious character (CP 5.181).

I begin by recalling that abduction takes an observed and surprising phenomenon as its premise and proposes a hypothetical explanation to account for it. The plausibility of such an explanation, therefore, depends on subsuming the specific features of the present instance under a general hypothesis that objects of that kind usually exhibit those features. Consequently, an acceptable abductive explanation of a surprising event must posit both a cause and a general rule that links that cause to the phenomenon. Within the Bayesian semantics of predictive processing, this logical structure finds its counterpart in the process of explaining away prediction error. The general rule corresponds to the prior distribution at higher levels of the generative model, which embodies the system’s expectations about the world. The cause, in turn, is the posterior estimate of hidden states that, under that prior, renders the sensory data most likely. Thus, Peirce’s logical account maps directly onto the core mechanics of predictive processing.

I maintain that what abduction sets out to explain is the surprising phenomenon, not the brute fact—whatever Peirce and the bulk of later writers happen to call it. Because the thing-in-itself (Ding an sich) is unknowable, only the empirical phenomenon shaped within the constraints of the *a priori* synthetic capacity is cognitively accessible. This stance is a Kantian premise rather than a self-evident truth, but it is adopted because Kant’s thought nurtured Peirce’s account of abduction and is compatible with the epistemological perspective of this paper^1^.

The feeling of surprise is the trigger of abduction. While Peirce provides the logical form of abduction, his account lacks a detailed cognitive mechanism for how surprise arises and is resolved. It is here that I introduce the Predictive Processing (PP) framework, as it supplies precisely this missing mechanistic account: the generation of a top-down prediction constitutes the formation of a hypothesis, and the minimization of prediction error through hypothesis revision is the process of abductive explanation. In this way, PP provides a neuro-computational account of how the brain continuously generates and tests hypotheses against sensory evidence, thereby implementing the very abductive logic that Peirce described (Barrett and Simmons, 2015).

Abduction may be intuitive and automatic or deliberate and controlled; only the latter counts as reasoning^2^. I therefore reserve abductive reasoning for its conscious, controlled variety and label its non-conscious, automatic counterpart abductive insight. The former is the deliberate formation and endorsement of hypotheses; the latter is their spontaneous, involuntary generation.^3^

It is important to address two related worries before I clarify this distinction: first, that abductive insight might collapse into Peircean habit, and second, that once habit is automatized it becomes indistinguishable from instinct. I answer both worries at once by referring to Peirce’s three-level map (CP 5.151–189): insight is a singular, content-specific flash that cannot be summoned or suspended; habit is a general disposition forged from once-voluntary acts and remains answerable to reasons in principle; instinct is phylogenetically fixed wiring that lies outside the space of reasons altogether. This three-way division secures the conceptual space needed to see how insight and habit interact yet remain apart. Insight provides the spark that nourishes habit; habit, even when automatized, retains its second-natural status and can in principle be re-voluntarized. The boundary is therefore preserved as a normative line: only second-nature processes are accountable on demand, whereas insight and instinct are not. Thus the sharp distinction stands.

In summary, abduction, as the logic of hypothesis generation, is fundamentally ignited by the experience of surprise or prediction error—an encounter with a phenomenon that contradicts our expectations. This foundational role of surprise positions abduction as a central cognitive operation not only in explicit scientific inquiry but also in the most basic processes of making sense of the world. It is to this most basic process—perception—that I now turn, arguing that the perception of the external world is the primordial domain of abductive explanation.

### 2.2 Perception

The classical phenomenological tradition, particularly in the work of Peirce, hinges on a fundamental distinction between two elements of perception: the percept (an involuntary, uncontrollable sensory image produced by external stimulation) and the perceptual judgment (a conceptual, meaning-assignment to that image based on background knowledge) (Luisi, 2006)^4^. My two-tier abductive model, introduced in the previous section, allows us to reframe this classical divide not merely as a phenomenological description but as the foundational manifestation of distinct abductive processes.

I argue that the Peircean percept is the paradigmatic instance of abductive insight. Its formation is the spontaneous, non-volitional generation of a hypothetical world-model in response to sensory surprise. Conversely, the perceptual judgment is the quintessential act of abductive reasoning, involving the conscious, deliberate categorization of the percept.

This mapping gains support when we examine the core phenomenological properties of each element. The percept is characterized by its irresistibility, meaning it forces itself upon our awareness independently of our will. It is also non-propositional, as it exists as a raw, qualitative gestalt prior to any conceptual analysis. In contrast, the perceptual judgment is inherently contestable. This is because its conceptual categorization is fallible and can be revised in light of new evidence or critical reflection. Furthermore, the perceptual judgment is propositional in nature, as it necessarily involves a claim about the world that can be articulated and evaluated.

This reconceptualization gains an additional layer of depth when viewed through Peirce’s semiotic lens. The percept, as an abductive explanation of sensory input, operates primarily through iconic signs (Firstness), capturing direct phenomenal quality. The perceptual judgment, as an abductive explanation of the percept, relies on symbolic signs (Thirdness), drawing on socio-cultural conventionality. The icon’s immediacy makes its formation an act of abductive insight; the symbol’s conventionality makes its operation an act of abductive reasoning^5^.

A potential objection to this abductive view of perception arises from anti-inferentialist theories, which argue that perceptual experience is direct and not mediated by unconscious inferences or hypotheses (e.g., Gibson, 1979). While this critique rightly targets overly intellectualist accounts, the Predictive Processing framework addresses it by positing that the requisite inferences are not conscious deliberations but automatic, sub-personal processes of prediction error minimization. This account reconciles the directness of phenomenal experience with the inferential nature of its underlying mechanism. Thus, the abductive model does not intellectualize perception but rather explains how its direct, immersive character is achieved through probabilistic inference.

More precisely, Gibsonian direct pickup is best read as a claim about the personal-level phenomenology of perception, not about its sub-personal causal story. Predictive Processing grants that no explicit, person-level deliberation occurs; nevertheless, the only way the system can keep the optic-array-coupling transparent is by continuously suppressing interoceptive and proprioceptive prediction error. That suppression just is the sub-personal abductive update. Empirical support comes from two directions: (i) psychophysical studies showing that artificially induced prediction-error (e.g., brief binocular rivalry perturbations) instantly alters what subjects report they see (Alink et al., 2010) and (ii) neuro-imaging work demonstrating that BOLD signals in early sensory areas track the precision-weighted prediction error rather than the raw stimulus (Murray et al., 2006). Consequently, the directness celebrated by ecological realism is not an alternative to but the phenomenological product of probabilistic abductive inference operating below the threshold of personal access. The anti-inferentialist objection therefore underdetermines the causal architecture of perception, whereas the abductive model explains both the felt immediacy and the measurable dynamics of sensory processing within a single formal framework.

### 2.3 Percept and abductive insight

I make my case in two moves: first, the percept is a plausible explanation of sensory manifold, so it is abductive; second, it is a felt quality devoid of truth-value and therefore outside volitional control, so it is insight.

My first step is to demonstrate that the percept is a product of abduction. Within the Predictive Processing (PP) framework, this abductive process is operationalized as the brain’s continuous generation of top-down predictions to explain away bottom-up prediction error (Swanson, 2016). The content of the percept is not an objective fact but an internal brain-centered construction. To mistake it for an intrinsic property of the world is a projection misattribution, which means the act of misattributing an internal construct to the external world. The core mechanism can be elucidated by Gibson’s notion of affordance (Gibson, 1979): the subject does not perceive objective properties but constructs relations of action-potential. For example, a cliff is perceived as dangerous because, in the very act of seeing it, the brain generates the production rule “if I step off, I fall.” This construction integrating fleeting sensory fragments via complex cognitive routines is first-person and self-evident, whereas the postulated entities, the body or the mind, are apprehended only through indirect presentation, introduced to render the percept natural. For instance, when one perceives glass as hard, the felt hardness is projected onto the glass, which in itself bears no such quality.

This abductive formation of percept confers two cardinal properties: intelligibility and wholeness. The property of intelligibility arises because the inherently meaningless sensory manifold conflicts with the brain’s constitutive drive to seek meaning, producing surprise. To eliminate this surprise, the agent must spontaneously generate an intelligible percept. Simultaneously, the property of wholeness is achieved because the sensory data alone is insufficient to form a complete perceptual experience, compelling the brain to supply additional top-down prior information to round out the perceptual episode.

My second step is to demonstrate that the percept is a felt quality devoid of truth-value: As Peirce puts it, “[It] does not stand for anything. It obtrudes itself upon my gaze; but not as a deputy for anything else, not ‘as’ anything. It simply knocks at the portal of my soul and stands there in the doorway” (CP 7.619). As a phenomenon that presents itself in immediate experience, yet-to-be-classified or related, it is the very embodiment of Peircean Firstness, which is understood here as a pure qualitative presence unconstrained by mediation. The Kantian *a priori* synthesis provides the necessary cognitive architecture that makes such an immediate, pre-conceptual quality possible at all; it is the formal condition for the manifestation of Firstness in experience. The following paragraphs will substantiate this claim from three angles, each drawing on a distinct theoretical framework to illuminate a different facet of the percept’s involuntary nature:

First, the formation of the percept is governed by the *a priori* synthetic capacity and therefore lies outside the control of the will. In Kant’s philosophy, the a priori synthesis has two interlocked senses (Kant, 1781/1787/1998): it is already built into human cognition before any experience, and it shows itself only in and through its influence on experience. Building on this Kantian ground, Gestalt psychologists have argued that human perception necessarily obeys laws such as proximity, similarity, good form, and continuity—laws that are culturally universal (Wertheimer, 1923). When a subject is confronted with a bouquet of flowers, for instance, he may lack the botanical knowledge required to name the species, yet without any prior learning he will automatically segregate the bouquet from the cluttered background as a single, coherent object. Thus, the incoming sensory excitation is inserted into a pre-given matrix of perceptual rules, and the percept is the product of that insertion.

Second, within Peirce’s semiotics, the percept is likewise beyond voluntary control^6^. The percept functions as an iconic sign that purports to denote some external object, but in Peircean phenomenology it belongs to the category of Firstness. Mental signs and physical objects are incommensurable (Gabriel, 2022); nevertheless, through a causal chain forged by the biological nervous system they enter an indexical relation that possesses brute compulsive force—this is Secondness. The qualitative feel of Firstness is independent of the subject’s will: one cannot, by sheer volition, alter the hue one sees. The indexical link between sign and object is constrained by the organism’s physiological makeup: under the relevant bodily conditions, a specific tissue lesion will inexorably hurt.

The two angles outlined above have established the percept’s involuntary character from philosophical and phenomenological perspectives. The third angle, grounded in the Predictive Processing (PP) framework, provides a neurocomputational account that operationalizes these insights. The Predictive-Processing (PP) framework dovetails with Peirce’s characterization of abduction: the initial perceptual prediction is the hypothesis, the comparison with sensory evidence is the test, and the updated perceptual content is the next hypothesis in the abductive cycle^7^. The sub-personal process of prediction error minimization instantiates the logical form of abduction at the system level: the generation of a new perceptual hypothesis (a revised prediction) that cancels surprise is functionally equivalent to inferring the most plausible explanation for the sensory data. I therefore cast the process in abductive form:

Observing a surprising phenomenon P;

if E were the case, P would be a matter of course,

Hence, there is reason to guess that E is plausible.

The process of percept formation proceeds accordingly:

Receiving an unexpected sensory input P;

were the perceptual prediction revised to E, P would be a matter of course,

Hence, there is reason to guess that revised prediction E is plausible.

The formation of a percept is a process of continuously generating, testing and revising perceptual predictions against external stimulation, an itinerary that falls squarely under the definition of abduction offered here. The mismatch between the sensory input and the anticipatory model is the very trigger that sets abduction in motion, while the adjusted prediction is the abductive explanation that resolves it. More precisely, the birth of a percept involves two intertwined phases: induction and abduction^8^. The iterative process of induction aims to minimize prediction error through model calibration. However, persistent prediction error creates the conditions that trigger an abductive leap to a new hypothesis. This abductive leap, by offering a more coherent explanation of the sensory data, retrospectively certifies the rationality of the inductive adjustments that led to it. The abductive aspect of percept is to highlight that the cerebral forecast confers meaningful intelligibility upon the newly arrived information, while its inductive aspect is to underscore that its very genesis depends on the continual juxtaposition of internal guess and external prompt.

In short, the percept prompts the subject to believe that an external object exists; that belief is recruited to explain the percept and is therefore abductive in character. Yet the emergence of the hypothesis is automatic and involuntary—it is not an episode of abductive reasoning but an abductive insight.

### 2.4 Perceptual judgment and abductive reasoning

This section analyzes perceptual judgment from two complementary perspectives: the phenomenological, which describes its intrinsic structure as a conscious experience, and the epistemological, which evaluates its status as a form of inferential justification. I will first establish its phenomenological structure and then argue that this structure embodies a genuine form of abductive reasoning.

I will argue for the intimate link between perceptual judgment and abductive reasoning in two steps. First, perceptual judgment is the very act of endowing the previously formed percept with meaning and explanatory significance; hence it is abductive in nature. Second, perceptual judgment can be brought under conscious control, yet it may also become automatized through habit. Even so, it remains in principle subject to conscious control. Even when operational outside awareness, it can be reintegrated into the arena of consciousness and brought under voluntary scrutiny. For this reason, it qualifies as genuine reasoning.

The first step is to argue that perceptual judgment is abductive because it is the very process of making the percept intelligible.

Perceptual judgment integrates a percept into a perceptual category, thereby endowing the former with meaning. The percept presents a raw, object-like unity; the judgment determines what that object is. It is fundamentally an operation of abductive reasoning, which employs signs—linguistic or otherwise, such as mental images—as its vehicles. Thus, this abductive process of categorization does not presuppose linguistic capacity^9^. In humans, the same act typically surfaces as naming. Naming carries two intertwined layers: (i) symbolization, i.e., the association of the percept with a sign that can stand for it (Wilson, 2023), and (ii) categorization, i.e., the placement of the object under a concept that bequeaths its defining attributes (Rosch, 1978). This classification can be cast as a tacit subject-predicate judgment. It is therefore evident that this subject-predicate structure of judgment does not narrow the scope of perception but is, in fact, the abductive move that first makes the percept intelligible.

Categorization can vary from rigorously defined to loosely practical. Crucially, regardless of the specific metric, every act of assigning a percept to a category is already an abductive explanation:

I have a percept P;

if P belongs to category C, then encountering P is a matter of course,

Hence, there is reason to believe P belongs to C.

Concrete case:

I have a percept P;

if P is an azalea, then seeing P is a matter of course,

Hence, there is reason to believe P is an azalea.

Having described the phenomenological structure of perceptual judgment, I now turn to its epistemological implications, which constitute the second step of the argument: from the standpoint of Peirce’s pragmatic semiotics, although perceptual judgments are usually taken to be pre-reflective and non-volitional episodes of cognition (CP 5.212), they nonetheless harbor an implicit reflective dimension. Precisely because it can be submitted to conscious scrutiny and control, perceptual judgment qualifies as abductive reasoning. This aligns with the core distinction introduced in Section 2.1: here, the term reasoning refers specifically to the conscious, deliberate formation of a hypothesis. It is precisely this capacity for voluntary control that distinguishes it from unconscious abductive insight.

Perceptual judgments possess propositional structure: to subsume a visual percept under a category X is already to assert “What I am seeing is an X,” thereby detaching the predicate X from the subject-term that indexes the experienced content (CP 7.631). Such judgments are therefore explanations of the percept in light of a conventional sign, a legisign (CP 5.54–56), and thus instantiate Thirdness in Peirce’s phenomenological scheme. Because legisigns are products of socio-cultural construction as well as vehicles for reflective activity, and reflection being typically conscious, perceptual judgments can be brought under conscious control.

The process of forming a perceptual judgment can be either an automatic routine or a deliberate undertaking. After a percept of a blossom has arisen, for instance, the subject might spontaneously subsume it under the category named flower; yet closer inspection can lead her to revise that classification and judge it to be an artificial bloom made of plastic. The first move is automatized, the second reflective. In other words, whenever categorization requires the conscious retrieval of background knowledge, the perceptual judgment is under conscious control; when the object is utterly familiar, the subject may classify it instantly and without reflection, and the judgment then escapes conscious governance.

Yet a judgment that escapes consciousness remains ontologically distinct from a mere percept. The generation of a percept is constrained by the subject’s innate physiological machinery; it is therefore inaccessible to conscious intervention. By contrast, even when a perceptual judgment is involuntary, its involuntariness derives from habits acquired after birth. This difference marks the fundamental boundary between abductive insight and abductive reasoning: the uncontrollability of the percept is rooted in the species-wide, evolutionarily given framework that underlies all conscious operation. The automatization of a perceptual judgment, however, is an efficiency-driven shortcut within the superficial strata of cognition, a second-nature sedimentation of formerly conscious activity whose inferential chain can in principle always be re-examined and revised. In cognitive psychotherapy, for example, negative beliefs or irrational cognitive schemas are treated as automatized thoughts consolidated through long-standing habit; if the therapist can help the client reflect deliberately on the legitimacy of each inferential step, the maladaptive pattern can gradually be ameliorated, thereby achieving therapeutic goals. In short, controllable reasoning may become automatized, and, conversely, automatized thought, once deliberately attended to, can be brought back under conscious control.

The proposed one-to-one mapping between percepts and abductive insight, and between perceptual judgments and abductive reasoning, aligns with the two-tier architecture of predictive processing. This alignment finds a mechanistic basis in the nature of the generative model that the brain is thought to maintain. As formalized by Friston (2010), this model is the joint probability distribution p(o,s)=p(s)·p(o∣s) , which constitutes the system’s complete “prior belief.” Its internal structure decomposes into two functional sub-components: the state prior p(s) , representing beliefs about hidden states, and the likelihood p(o∣s) , encoding the probability of observations given those states. This foundational mathematical structure is populated by specific prior expectations, which Clark (2013) categorizes into two tiers: innate priors, encoded in the genome and refined by evolution, and learned priors, etched by a lifetime of experience. Building on this, the present paper proposes that strong predictions, stemming from these innate cognitive dispositions, generate the percept as an abductive insight. Conversely, weaker predictions, arising from empirically entrenched routines, shape the perceptual judgment as a form of abductive reasoning. Thus, the entire perceptual process can be understood as a hierarchical Bayesian inference operating on the components and of the generative model, where different types of priors contribute to distinct abductive stages.

Under the constraint of the strong model, the agent first generates weak priors, which in turn feed into the formation of perceptual predictions in an abductive hypothesis-formation stage, while incoming sensory data then modulate these predictions in a hypothesis-testing stage. Repeated predictive failure prompts the agent to reorganize or revise the prior model. Across evolutionary time, the strong model itself is gradually reshaped through inter-generational adaptation, yet for any single agent it remains immutable.

Having established perception as a hierarchical abductive process driven by the need to explain sensory surprise, specifically exteroceptive prediction error, we now generalize that this same logic extends seamlessly from the external domain of exteroception to the internal domain of interoception. Just as the visual system generates abductive hypotheses to account for an unexpected sight, the emotional system generates hypotheses to account for unexpected internal physiological changes.

## 3 Abduction and the construction of emotion

Building on the unified model that aligns perception with abduction, this section extends the same theoretical dyad to the interoceptive domain of emotion. Within the predictive processing paradigm, surprises in the body’s state, known as interoceptive prediction errors, trigger an abductive process. I examine this process through the lens of Barrett’s Theory of Constructed Emotion (Barrett, 2006), which posits that emotions are built from two components: core affect and emotional categorization. This division directly instantiates the two-tier abductive structure. Core affect functions as abductive insight, an uncontrolled registration of internal perturbation, while emotional categorization acts as abductive reasoning, inferring the most plausible cause, such as anger or fear, to explain the feeling. The following sections detail this argument.

### 3.1 Core affect and abductive insight

The core insight of the Theory of Constructed Emotion can be traced back to the peripheral theory of emotion originally proposed by James (1884). According to this view, emotional experience arises not from the perception of an external stimulus itself, but from the bodily changes—both involuntary physiological responses and voluntary skeletal actions—that follow it. In other words, we do not tremble because we are afraid; we are afraid because we tremble. Building on this foundation, Peirce (CP 5.292–294) and Damasio (2010) offer a more refined perspective. They argue that unconscious biochemical shifts are the primary drivers of emotional feeling. Importantly, since skeletal actions themselves induce further physiological changes, a causal loop emerges: physiological activity gives rise to emotional experience, which in turn motivates bodily action; that action then generates new physiological changes, perpetuating the cycle. Thus, physiology, emotion, and behavior are not linearly linked but dynamically interwoven.

Based on the Theory of Constructed Emotion, core affect is the subject’s integrated perception of autonomic, hormonal, visceral, and immune signals generated within the body (Costa et al., 2022). From this perspective, core affect is, in fact, a percept about the organism’s internal physiological state; since percepts are abductive insights, the generation of core affect counts as an abductive insight that extracts an affective pattern from bodily conditions. I propose that core affect encodes affordances by registering both internal bodily changes and the action possibilities that external objects have previously offered the body. In doing so, it integrates physiological signals with situational cues. Core affect thus operates on two levels: as emotion it propels mental life; as abduction it supplies cognition with affective patterns. This gives core affect a hybrid character that is at once affective and cognitive.

This abductive insight, however, can be analyzed on two distinct levels. On an epistemic or functional level, it is highly effective and successful—it is a “genuine” insight in that it provides a pragmatically useful model of the body’s state that guides action. On a separate ontological level, however, the way this model is typically interpreted by the conscious subject involves a mis-identification born from the subject’s projection of interoception onto the hypothetical entity called mind. This mental substance is pressed into service as a bearer: the subject’s avowal “I feel anxious” is logically the predication of an affect-property to the mind-subject. Though illusory, the mind-entity is retained because its abductive explanation of visceral signals is pragmatically useful.

From a Predictive Processing perspective, this process can be re-described in more formal terms: when the interoceptive signal F_1_ is not accounted for by the current core-affect model E_1_, the agent automatically minimizes prediction error. Two routes are open: 1. Keep E_1_ and allow it to trigger biochemical changes that produce an F_1_-congruent bodily state; 2. Let F_1_ drive the rapid formation of a new core affect E_2_ that better fits the incoming signal. Route 2 instantiates abduction: a new percept (E_2_) is generated to explain the surprising interoceptive datum. Yet because this update is effortless and unconscious, it is not a reasoning but an abductive insight.

To concretely illustrate the formation of core affect as an abductive insight, consider the initial physiological upheaval of fear. Upon encountering a sudden, looming shadow, the body undergoes a rapid perturbation: the heart pounds, muscles tense, and attention narrows. This cascade of interoceptive and proprioceptive data is, in itself, a surprising and unstructured sensory event. The brain’s automatic, non-conscious response is to generate a unified core affect—a palpable feeling of arousal with a negative valence—as an initial hypothesis to explain this bodily surprise. This is the abductive insight in its primordial form: the spontaneous generation of a felt, affective quality (the core affect) in direct response to sensory prediction error. It is the raw material of emotion, prior to any conceptual labeling as fear.

### 3.2 Emotional categories and abductive reasoning

This sub-section elaborates on the second tier of emotional abduction: the formation of emotional categories as abductive reasoning. The argument proceeds in four steps. First, I establish the abductive nature of categorization itself. Second, I examine its dual functions in communication and self-regulation. Third, I explore how these functions are refined through emotional granularity and shaped by cultural context. Finally, in the next sub-section I shall formalize this entire process using a set-theoretic model to clarify its logical structure.

Emotional categories^10^ represent the cognitive subject’s contextualized explanation of interoceptive signals. This explanation is shaped through a dialectical interaction between two factors: experiential ontogeny, defined as the lifelong development of emotional concepts through lived experience; and functional constraints, understood as the adaptive pressures that prioritize explanations serving survival and social needs^11^. The interplay of these factors means that emotional categories are neither purely subjective constructions nor rigid biological reflexes, but adaptive cognitive patterns formed at the intersection of personal history and species-typical needs.

Within this framework, emotional categories are conceptualized as the brain’s best guess, the formation of which relies on the integration of interoception, past experiences, and socio-cultural context. The process of emotional categorization—where a subject names, classifies, and conceptualizes their core affect—is analogous to the process of perceptual judgment, which involves integrating a manifold of sensations and conceptualizing it. Since perceptual judgment is abductive in nature, the formation of emotional categories is likewise abductive: if an individual names a vividly experienced core affect as a specific emotional state, that experience becomes plausibly explained, thereby providing a reason to believe one is indeed in that emotional state.

This abductive process of categorization can be illustrated by returning to the example of the core affect generated in a moment of threat—the visceral feeling of arousal with a negative valence. The brain abductively categorizes this feeling as fear: “I feel this intense alarm and dread (core affect C); if I were in a state of fear (hypothesis A), then this physiological state would be expected; hence, there is reason to conclude I am in a state of fear.” This act of naming is the emotional analogue of perceptual judgment, transforming raw core affect into a specific, actionable emotion through abductive reasoning.

The construction of emotional categories is fundamentally guided by survival needs, grounded in an embodied and situated view of cognition. These categories serve core survival functions, primarily by enabling communication to coordinate with others and self-regulation to guide adaptive action. However, the efficacy of these functions is not uniform across individuals; it is modulated by the individual’s level of emotional granularity, which refers to the level of refinement in a subject’s conceptualization of their own core affect (Fox and Riconscente, 2008). Higher granularity enables more precise abduction, allowing for finer differentiation of core affect^12^. This precision, in turn, leads to more targeted communication and more adaptive self-regulation strategies, ultimately fulfilling the adaptive purpose of emotional categorization.

First, I will zoom in on the communicative payoff: emotional categories turn bodily states into public signals that coordinate threat, care, or opportunity within the group. The primary value of this abductive categorization becomes evident in its pragmatic functions. Core affect exists solely within the subjective consciousness of the individual, characterized by its privacy and first-person nature. To achieve effective information transfer, the speaker encodes pre-reflective bodily awareness into culturally shared emotional categories, while the listener engages in analogical simulation based on similar experiences from their own experiential repertoire. From a psychodynamic perspective, this transmission of emotion fosters an empathic experience. From the standpoint of cognitive philosophy, this process constitutes an embodied form of analogical reasoning: when attempting to understand the speaker’s core affect, the listener unconsciously matches the speaker’s linguistic descriptions against their own past experiences that have been categorized under the same emotional label. This projection of one’s own feelings onto others forms the psychological basis for empathy, though it inevitably involves some degree of distortion.

Second, I will show how the same categories loop back into the organism as fast self-regulatory scripts, tightening or loosening physiological resources to match the here-and-now demands of survival. Self-regulation is essentially a process of symbolic reconstruction. When core affect is categorized under a specific emotional label, it effectively constitutes a process of cognitive development, wherein vague affective experiences are assimilated into conceptual tools acquired through social learning. This process embodies the fact that concepts function as tools: different emotional labels imply distinct action strategies. For instance, parsing a racing heart and narrowed vision as fear recruits the script flee-hide-quiet, shunting blood to legs and widening airways for escape; parsing the same surge as anger instead scripts chest-forward-attack, vasodilating the arms and sharpening strike precision for confrontation—two diverging bodily itineraries launched by a single categorical flip.

Thus, the very communicative and self-regulatory dividends just described hinge on a prior, moment-to-moment abduction: only if the organism can flexibly re-label the same visceral surge as fear in the dark alley or anger under public gaze can flight or confrontation be launched in time. In short, the two functions are viable only because the mapping from core affect to emotional category is intrinsically dynamic and context-bound.

The dynamism manifests in the fact that even when the same individual experiences similar physiological arousal, their emotional categorization can fluctuate due to differences in situational cues, immediate cognitive appraisals, and memory retrieval. In short, this variability corroborates the core proposition of the theory of constructed emotion—that emotional experience is not a simple readout of fixed physiological patterns but rather a real-time explanation, by the brain, of ambiguous bodily signals based on the currently available conceptual system.

Context dependence signifies that cultural background profoundly shapes the pathways of emotional explanation through linguistic systems and conceptual frameworks. Different linguistic cultures develop unique emotion lexicons, and these culture-specific emotional categories constrain the scope of people’s explanations^13^. For example, cross-cultural research indicates that East Asian cultures tend to express emotional distress more through somatic symptoms, whereas Western cultures are more inclined to express it through psychological symptoms (Ryder et al., 2008; Choi, 2016). This divergence between psychologization and somatization may stem from deeper cultural cognitive styles. The diversity in cultural cognition reveals that emotional categories are embodied constructs steeped in cultural influence. When a Western individual might describe feelings of depression as a “wounded soul,” a Chinese individual might employ the metaphor of tagnation of liver qi from Traditional Chinese Medicine. Such differences in explanation represent the embodiment of distinct cultural worldviews within the affective domain. Each interpretive path carries specific ontological commitments and epistemological presuppositions, collectively constituting the pluralistic landscape of human emotional understanding (Kirmayer, 2015).

Mirroring the process in perception, the categorization of core affect into a specific emotion creates a parallel feedback loop, whereby the categorization subsequently modulates the core affect itself. First, once core affect is subsumed under an emotional category, the resulting belief that one is experiencing that emotion drives the subject to engage in category-consistent behaviors, which then modify physiological states and consequently alter the core affect (Barrett, 2017a; Barrett, 2017b). Second, the same emotion label can correspond to heterogeneous core affective states (Hoemann et al., 2019), while identical core affect may be categorized under different emotion labels. Crucially, once an emotion label is established, its associated semantic network becomes activated, thereby reshaping subjective experience. For instance, if an agent defines her current state as “depression,” she may subsequently exhibit depression-typical behaviors implied by this conceptualization. These behaviors then intensify the core affect, while the semantic constellation carried by the term depression (e.g., helplessness, suicidal ideation) becomes activated, potentially creating a self-reinforcing cycle. This bidirectional process exemplifies how emotion concepts, as symbolic tools, participate in both the explanation and constitution of emotional reality. Thus, what looks like mere labeling is in fact an act of abductive reasoning: the subject retro-dicts the most plausible emotional cause for the felt perturbation.

In summary, emotional experience comprises both core affect and constructed emotion. The formation process of the former is analogous to that of the percept, thus belonging to abductive insight; whereas the formation process of the latter is analogous to that of the perceptual judgment, thus constituting abductive reasoning.

### 3.3 A formal (set-theoretic) model of emotional construction

The preceding analysis has outlined a complex process involving physiological states, core affect, and conceptual categorization. To formalize these relationships and capture the logical structure of emotional construction in a precise manner, I now introduce a set-theoretic model. This formulation serves to crystallize the distinction between abductive insight (the mapping from body to feeling) and abductive reasoning (the mapping from feeling to concept), while providing a rigorous framework for understanding the underdetermination of emotional experiences by physiological states.

I can formalize this as follows: To clarify the argument, let us posit that all physiological states of a subject constitute Set A, all core affects constitute Set B, and the subject’s cognitive delineations of these core affects—that is, the results of naming, classifying, and conceptualizing them—constitute Set C. Let the formation process of core affect be function F from A to B, and let the cognitive delineation of core affect be function G from B to C. Thus, for any b∈B, there exists an a∈A such that b = F(a); similarly, for any c∈C, there exists a b∈B such that c = G(b). Consequently, for any c = G[F(a)], there is a corresponding a∈A. This means that for any named emotional category, I can always identify a specific physiological activation state that corresponds to it.

The mapping from Set A (physiological states) to Set B (core affect) is non-injective. This many-to-one geometry has a profound theoretical implication: it means that the same core affective state (an element in B) can be covered or explained by distinct emotion concepts (various paths through function G). This, in turn, creates the essential space for cultural and contextual modulation—a possibility that is central to the theory of constructed emotion. In this sense, non-injectivity serves as the mathematical signature of constructivism.

This many-to-one mapping comes from two limits of the senses. First, some bodily changes are too weak to cross the absolute threshold, so they never reach the level of feeling; no core affect is triggered. Second, the difference threshold is not fine enough: two clearly different bodily signals can land on the same felt state. The same loss of detail happens again when the smooth range of feelings is sorted into separate emotion words; how finely one splits that range is what we call emotional granularity.

This model formalizes two core ideas of constructed emotion. Because both F and G are many-to-one, the same body state can end up as different emotions when context shifts the word we choose. That is the math behind emotional plasticity. How finely we split core affect into words is simply the resolution of G, called emotional granularity. With the links among physiology, core affect, and emotion categories now laid out, the next step is to ask what this means for everyday emotion regulation. The following section turns to that practical question.

### 3.4 Implications for the practice of emotion management

Viewing emotion through the lens of abduction provides practical guidance for maintaining emotional health. This paper’s two-tier abductive model frames emotion regulation along two matching routes: one works by abductively engaging core affect directly through lived experience; the other by sharpening emotional granularity, which amounts to splitting the felt sense into finer, more precise names. The first pathway, which targets abductive insight, leverages the fact that core affect is grounded in physiological states. Therefore, the approach of improving core affect by altering physiological states gains deeper theoretical support. The second pathway operates at the level of abductive reasoning, treating every emotion word as a personal theory about one’s own core affect. When that theory is vague or negatively framed, it narrows attention, warps expectations, and locks the individual into problematic response patterns. By weaving physiology and conceptual labeling into the same abductive cycle, the model thus blurs the traditional line between mind and body, helping to mitigate the influence of Cartesian mind–body dualism and challenge dualistic assumptions that strictly separate mental and physiological interventions. A dual-route approach is therefore recommended:

The first route involves direct experiential engagement with core affect. This approach entails attending to the raw feeling itself. It aims to decouple the sensation from its habitual conceptual explanations, thereby reducing automatic cognitive appraisal and cultivating the direct, non-inferential awareness that characterizes abductive insight. This practice is the philosophical cornerstone of mindfulness therapy (e.g., Kabat-Zinn, 2003). For example, rather than categorizing anxiety as a “negative emotion,” the individual learns to observe and experience the underlying sensations, such as a racing heart or sweaty palms. Studies indicate that this practice enhances emotion regulation ability by shifting the meta-cognitive stance from self-judgment to self-awareness (Holzel et al., 2011; Salem et al., 2025).

The second route involves enhancing emotional granularity through refined labeling. This strategy entails the deliberate process of generating and testing more precise explanatory hypotheses for the core affect. Individuals with high emotional granularity can deconstruct vague feelings into finer-grained categories, which inhibits overly generalized negative explanations (Bonar et al., 2021). Research shows that a refined emotional vocabulary allows individuals to predict their emotional trajectory more accurately, thereby reducing defensive reactions caused by cognitive biases (Kashdan et al., 2015). Furthermore, high granularity is associated with more differentiated physiological response patterns, such as in heart rate variability (Hoemann et al., 2021).

When both tracks are trained concurrently, with body work preceding language work, participants show significantly larger reductions in negative affect and greater gains in granularity than after single-track programs (Farb et al., 2015). This combined effect can be pictured in terms of image quality: Low-granularity emotion construction is akin to a low-resolution image, which can only vaguely capture the contours of an emotion. Pathological emotion construction, on the other hand, is like pixel misalignment caused by a sensor malfunction, resulting in systematic distortion. The first approach mentioned earlier bypasses conceptual representation by enhancing interoceptive awareness, similar to a photographer putting down the camera to observe the subject with the naked eye. The second approach improves the quality of the “image” by refining the network of emotional categories, which is equivalent to upgrading the optical components and image processing algorithms.

Therefore, effective emotion regulation can be understood as the flexible application of interventions that either alter the percept through abductive insight, reshape the judgment through abductive reasoning, or combine both. This abductive framework provides a unified theoretical basis for understanding the mechanisms of diverse therapeutic approaches.

## 4 Conclusion

This paper has proposed and defended a two-tier model of abduction, distinguishing between spontaneous abductive insight and deliberate abductive reasoning. The primary achievement of this model is its resolution of a fundamental tension within Peirce’s framework by demonstrating how perception and emotion can be understood as forms of abduction while respecting the distinction between involuntary and voluntary processes.

By operationally defining the contested concepts of consciousness and controllability, I have integrated Peircean logic with the mechanistic account of Predictive Processing and the psychological specificity of Barrett’s Theory of Constructed Emotion into a cross-level explanatory framework, providing a coherent account of the mind as a unified inferential engine. The explanatory power of this unified framework is demonstrated across two key domains, namely perception and emotion: In the domain of perception, I have argued that the formation of the percept constitutes abductive insight, while the perceptual judgment constitutes abductive reasoning. In the domain of emotion, I have demonstrated that emotion is constructed through an abductive pathway which begins with the generation of core affect as abductive insight and culminates in its categorization into a specific emotional category as abductive reasoning, in line with Barrett’s theory of constructed emotion.

By integrating Peircean logic with contemporary predictive processing and constructionist theories, this research provides a cross-level explanatory framework that bridges philosophy and cognitive science. This framework allows us to resolve the original tension by recasting Kant’s passive synthesis as the bottom-up prediction error in PP and Peirce’s abduction as its top-down predictive hypothesis. I thus demonstrate that perception and emotion qualify as forms of abduction not in spite of, but precisely because of their dual nature: they are simultaneously automatic in their initial insight and reason-responsive in their subsequent conceptualization. In a wider sense, the abductive perspective advanced here does more than illuminate specific cognitive processes; it offers a powerful new lens for understanding the profound unity and complexity of human cognition as a whole, with implications for both theoretical inquiry and practical application.

## Data Availability

The original contributions presented in the study are included in the article/supplementary material, further inquiries can be directed to the corresponding author/s.

## Footnotes

1 PP likewise posits an inaccessible boundary: prediction error can only be inferred, never directly accessed; Kant’s phenomenon/thing-in-itself divide is recast within PP as the irreversible asymmetry between internal hidden states and external generative parameters.

2 Abductive reasoning is not abductive inference. Peirce explicitly separates the two: “By reasoning I mean the conscious, controlled passage of thought from one proposition to another; by inference the abstract logical relation that such a passage instantiates” (CP 5.108). In this paper I preserve that distinction while treating “abduction” and “abductive inference” as co-extensive: both cover the same trajectory from surprise to hypothesis, but “abductive inference” foregrounds the procedural aspect, whereas “abduction” names the whole epistemic unit that can be examined as an object of inquiry. Thus abductive reasoning designates the consciously governable psychological act, and abductive inference designates its logical abstraction.

3 My coinage “abductive insight” is not Peircean verbatim, yet it is motivated by a classical contrast: Aristotle distinguishes hexis (a settled, recallable disposition) from kairós (the singular, unrepeatable moment of insight). Insight, in this lineage, is the one-off flash; habit is the retrievable capital. Kant furnishes a parallel when he separates passive synthesis (which delivers the sensory given) from active judgment (which subsumes it under concepts). Hume, too, opposes custom (repeated association) to sudden belief-revision (the burst of surprise (Magnani, 2009). Peirce re-engineers these poles into abductive insight versus abductive reasoning, and Magnani’s “tacit abduction” provides the contemporary bridge. my two-tier model merely makes the polarity explicit and operationally tractable within present-day cognitive science.

4 This distinction has conceptual precedents, such as Hume’s separation of sensory impressions from the ideas that are their copies (Hume, 2000), and engages with Kantian concerns regarding the noumenal. However, Peirce’s robust realism and semiotic framework mark a fundamental departure from Hume’s nominalism. Thus, while Hume provides a useful point of departure, Peirce’s account is distinct in its philosophical commitments.

5 Peirce divides every appearance into three layers. Firstness is sheer quality, the flash of redness before it is pointed at or named, simply shining on its own. Secondness is the brute shock of encounter, the way pain announces itself when a knee hits the table, an indexical jolt that cannot be denied. Thirdness is the realm of rules and symbols where we borrow words, concepts, or images to place that redness under “fire” or that pain under “danger,” turning the moment into shareable meaning. Within predictive processing, Firstness maps to the sensory icons generated by high-level priors, Secondness to the bottom-up surge of prediction error, and Thirdness to the later symbolic redescription of that error aimed at reducing long-term surprise.

6 I engage here in a theoretical reconstruction of Peirce’s semiotics, interpreting its concepts through a modern cognitive-scientific lens to bridge philosophy and cognitive science. This approach aims for a productive dialogue with Predictive Processing, not a purely historical explanation.

7 This is not merely a metaphorical analogy but a claim about mechanistic implementation. That is, the computational process of prediction-error minimization is argued to be a constitutive mechanism that physically realizes the logic of abduction.

8 Here “induction” is used in a broad sense: the brain’s repeated calibration of a hypothesis—i.e., the predictive model—across many sensory samples, extracting their shared structure. It is the Bayesian updating of beliefs aimed at minimizing prediction error, not the enumeration-to-universal-proposition move of classical logic.

9 This claim is supported by two key clarifications regarding the two-tier abductive model. First, the logical form of categorical judgment (subsuming a particular under a general) is not dependent on language. It is operational in non-linguistic cognition (Barsalou, 2008), as seen when a chick pecks at a grain or an infant recognizes an affordance. Second, the automatic generation of the perceptual hypothesis (abductive insight) is logically and temporally prior to any conscious judgment (abductive reasoning). While critical reflection can later evaluate the hypothesis, the hypothesis must first be present as a meaningful Gestalt for evaluation to occur. Together, these points underscore the distinct yet complementary roles of involuntary insight and voluntary reasoning in perception.

10 The term “emotional categories” corresponds to what Barrett refers to as “emotions as categories” or “constructed emotions” (Barrett, 2017a; Barrett, 2017b). For the sake of clarity and consistency in this discussion, this paper will uniformly use the term “emotional categories.”

11 Experiential ontogeny refers to the lifelong process by which an individual gradually constructs an understanding of emotions through continuous feeling and experience. Emotional categories are not innate but are cognitive patterns formed through maturation, life experiences, and cultural learning. This process involves perceiving internal physiological signals, observing the external environment, and reflecting on past experiences, whereby the individual learns to classify primitive physiological sensations, thereby forming a cognitive framework for specific emotions. Functional constraints emphasize that the formation of emotional categories is guided by adaptive goals. The brain’s explanations are optimized to meet demands of survival, adaptation, or social interaction. Thus, these categories are not merely explanations but must serve adaptive goals. For instance, interpreting a racing heartbeat as fear (prompting avoidance) rather than anger (prompting confrontation).

12 Within the theory of constructed emotion (Barrett, 2017a), emotional granularity refers to the level of refinement in a subject’s conceptualization of their own core affect. A higher level of refinement corresponds to higher granularity, and the precision of this emotional naming is positively correlated with the efficacy of the aforementioned functions (communication and self-regulation). The greater an individual’s vocabulary of emotion terms, the higher their emotional granularity, and consequently, the stronger their cognitive capacity to differentiate between various states of core affect.

13 A prime example is the Chinese concept of shànghuǒ, a notion from Traditional Chinese Medicine referring to a state of internal heat imbalance. It encompasses a range of physical and affective symptoms (e.g., sore throat, agitation) that have no single, direct equivalent in the English lexicon, demonstrating how emotion concepts are embedded within specific cultural and medical worldviews.
